# Is networking different with doctors working part-time? Differences in social networks of part-time and full-time doctors

**DOI:** 10.1186/1472-6963-8-204

**Published:** 2008-10-04

**Authors:** Phil JM Heiligers, Judith D de Jong, Peter P Groenewegen, Lammert Hingstman, Beate Völker, Peter Spreeuwenberg

**Affiliations:** 1 NIVEL - Netherlands institute for health services research, PO Box 1568, 3500 BN Utrecht, The Netherlands; 2 Utrecht University, Faculty of Social sciences, PO Box 80.140, 3508 TC Utrecht, The Netherlands

## Abstract

**Background:**

Part-time working is a growing phenomenon in medicine, which is expected to influence informal networks at work differently compared to full-time working. The opportunity to meet and build up social capital at work has offered a basis for theoretical arguments.

**Methods:**

Twenty-eight teams of medical specialists in the Netherlands, including 226 individuals participated in this study. Interviews with team representatives and individual questionnaires were used. Data were gathered on three types of networks: relationships of consulting, communication and trust. For analyses, network and multilevel applications were used. Differences between individual doctors and between teams were both analysed, taking the dependency structure of the data into account, because networks of individual doctors are not independent. Teams were divided into teams with and without doctors working part-time.

**Results and Discussion:**

Contrary to expectations we found no impact of part-time working on the size of personal networks, neither at the individual nor at the team level. The same was found regarding efficient reachability. Whereas we expected part-time doctors to choose their relations as efficiently as possible, we even found the opposite in intended relationships of trust, implying that efficiency in reaching each other was higher for full-time doctors. But we found as expected that in mixed teams with part-time doctors the frequency of regular communication was less compared to full-time teams. Furthermore, as expected the strength of the intended relationships of trust of part-time and full-time doctors was equally high.

**Conclusion:**

From these findings we can conclude that part-time doctors are not aiming at efficiency by limiting the size of networks or by efficient reachability, because they want to contact their colleagues directly in order to prevent from communication errors. On the other hand, together with the growth of teams, we found this strategy, focussed on reaching all colleagues, was diminishing. And our data confirmed that formalisation was increasing together with the growth of teams.

## Background

Teams of medical specialists are involved in several changes, such as technical developments, hospital mergers and the integration of doctors working part-time. Part-time working is not common in medical practices, although individual specialists stress the need for reduced working hours [1-5]. One of the problems in realising part-time work is the underlying discussion about responsibility for the continuity and quality of care. It has been argued that a minimum of hours worked is necessary in order to prevent patients and colleagues suffering from undesirable consequences, such as a lack of information or communication errors [6]. For professionals in medical care, consultation and communication between colleagues is essential to provide high standards in quality of care [7]. Most transfer of information is provided in social relations within informal networks at work. In this article we focus on the consequences of part-time working within those informal work-related networks. Part-time doctors with reduced working hours are limited in the amount of time they can invest in these networks in comparison to full-time workers.

Another aspect is: What type of informal work relationship is important either for part-time or full-time doctors? In work relationships of professionals, such as medical specialists, mainly three types of networks are important. Firstly, information about all kinds of work issues is transferred in communication networks. Secondly, specific professional issues are discussed in consulting networks in order to support each other. And, finally, especially in medical professions relationships of trust are important, because on the one hand confidential information about patients should be treated very carefully, and on the other hand, working in a joint partnership is based on confidential relationships with team members, which can be found in trust networks.

In addition to the type of network, the characteristics of the networks might also differ between part-time and full-time doctors. In the first place the size of informal work-related networks will be studied, because it is important to have enough network members to gain optimal benefits [8]. Furthermore, it is important how often doctors use their network relationships and how intensive relationships are. And, finally, especially for part-time workers, it is important to build their network contacts efficiently, meaning that part-timers can be reached easily by others without the necessity to have personal contact.

In summary, we want to answer the question:

• *What are the differences in informal work- related networks of part-time and full-time working doctors and to what extent are these differences related to individual characteristics and characteristics of the team as a whole?*

We will answer this question with regard to the work situation of medical specialists in the Netherlands.

### The organisation of part-time work in self-employed partnerships

In the Netherlands most medical specialists work in self-employed teams, financially independent from hospitals. Doctors with the same specialist background select their partners for specialist teams. So they work in partnerships of independent professionals, an organisational structure in which partners are mutually dependent, and defend their own interests as well as common resources [9]. They work as equals with the same responsibilities for quality of care, production and joint income. There is no formal hierarchical structure and all business decisions are discussed with all members of the team. This creates an organisational difficulty for the introduction of part-time work [1]. Rationally, it is not difficult to relate the lower investments of part-time workers by pro rato income and division of tasks. However, the basic idea about sharing responsibilities and decisions within partnerships can be frustrated. Full-time workers can perceive their position as overloaded with extra work in terms of responsibilities [2]. Part-time doctors can experience a loss of control over information and as a consequence expect a less influential position concerning core decisions within the partnership.

#### Theoretical background

The independent partnerships of doctors in the Netherlands are not very formal or structured. So communication and relationships are mainly based on informal contacts and networks, which play an important role in these small teams of self-employed partners. In this article we focus on these networking relations at work, both self-organised and informal. Informal networks are mostly voluntary that is between members of the organisation who discuss issues to do with the organisation unofficially [10]. To answer our research question we turn to complementary theoretical explanations for building social relations such as "the opportunity to meet" [11] and "building social capital" [12,13]. Both approaches will be discussed below and we will point to the specific position of part-time workers. For doctors working in self-employed partnerships, working part-time is the result of negotiations with the other partners. Negotiations are focussed on the individual contribution to the total package of services the partnership has to provide for the hospital. Full-time equivalents (fte) rule the division in tasks and contributions and also the division of income. The amount of working hours is not used to define part-time working. Regardless some diversity between specialties it was found that full-time working doctors (1.0 fte, 100%) on the average work about 50 hours weekly [1,2,5]. Part-time partners, working 0.8 fte or 80%, will than work about 40 hours a week.

### The meeting argument and part-time working

The development of social relations is basically explained by the meeting argument of Blau [11]. People need the opportunity to meet in order to build social relations. Working together is one of the social contexts or meeting points where social relations develop [14]. On this point part-time workers are restricted because they spend less time at work in comparison with full-timers [15,16]. In line with this "meeting" argument, it can be expected that part-time doctors have less opportunity for daily contacts.

Another aspect related to the opportunity to meet is the size of teams. It can be expected that team size will increase with the number of part-time doctors, because a team will need more individuals to do the same amount of work. Firstly, larger teams coincide with increased opportunity to contact different colleagues, but part-time doctors are restricted in time, implying a limitation in the size of personal networks. Secondly, the frequency of contacts will decrease because of the limitation in time. The larger a team is, the lower the frequencies of contacts with each other will be [17]. We expect that frequency of contacts is not only limited for part-timers but also for full-time doctors working in teams with part-timers.

### Building social capital and part-time working

In social capital theory, building social relations and personal networks are seen as investments. Individuals enlarge their social capital by investing in others [8]. The amount of social capital is not only based on individual efforts or investments, but also relies on the numbers of others in personal networks and the resources they can, and will, offer.

The amount of time an individual spends at work is one of the resources for building a personal network as an investment in social capital. Due to time restrictions part-timers will invest less in building social capital and consequently will have fewer resources at work [12]. As mentioned above three types of networks are important at work: communication, consulting and trust networks.

In **communication **networks, we find team members who *talk to each other about work *on a regular basis [18]. Since part-time doctors are less present they obviously participate less than full-timers in this kind of regular talking.

Central to the network of **consulting **relations are the prominent players in a team, of whom others are dependent for solving problems or getting access to technical information [18]. Connections in this type of network provide part-time doctors with relevant *information *for *decision-making *within their partnership. Part-time workers will mainly seek instrumental support, such as advice in consulting networks. Even less strong or less frequent network relations are sufficient for receiving this kind of support [12,13].

The network of **trust **gives insight into which team members would share *confidential information and feedback *and who gives support to someone else in crises [18]. Trust is inevitably important within any partnership comprising a company of equals. All doctors in a partnership are dependent on the investments and support of colleagues [19]. It can be expected that doctors working part-time and full-time do not differ in sharing confidential matters at work. Trust and sharing confidential matters is a condition of participation in a partnership. The same argument holds for social-emotional relations within a partnership. These relations imply talking about personal questions, which is related to sharing confidentiality.

Furthermore, for doctors working part-time, efficiency might be very important. In terms of social capital they might prefer to invest in colleagues who have many connections with others as this is an efficient way for part-time workers to be reached by others. Non redundant contacts offer more information that is more often new [20]. In other words: seeking contacts among powerful or coordinating colleagues, who are in the position that they are contacted by many others, offers more information compared to the information an isolated colleague could provide us with.

### Summary of hypotheses

Based on the arguments above the following **hypotheses **are formulated:

1. In line with the "meeting" argument, it can be expected that the size and frequency of contacts in informal work-related networks are less for part-time doctors, compared to full-timers; full-time doctors in teams with part-time workers will be limited only in frequency of contacts.

2. In terms of social capital, part-time doctors are restricted in how far they can offer and receive resources. In order to broaden their resources of information they will choose relations with colleagues who have many contacts in the team. In technical terms, part-time doctors will invest in "high reach efficiency" in informal work-related networks;

3. For building social capital it can be expected that part-time and full-time doctors do not differ in regard to the sharing of confidential matters and social- emotional relationships.

### Further team and individual influences

Apart from influences in line with the theoretical background regarding the meeting argument and building social capital a few other aspects influence informal networks. Firstly, the type of specialty may influence relations in doctors' networks. In our study we included internists, surgeons and radiologists. The structure of their work environment differs, because some specialties involve many patient contacts (internists), others have restricted location tasks (surgeons in the operating room), and again others are working more with technical equipment (radiologists) and can easily leave their location. Opportunities to communicate in these specialties differ. So the type of specialty might influence the frequency of contacts and maybe the number of relationships.

Furthermore, gender seems to have a general influence on building networks at work in favour of men [21-23] and also influences related to age and tenure or years in partnership, have been found. Age seemed to be more important because being older can cause isolation [24,25].

### Informal networks at work and formal structures

Teams are not only regulated by informal contacts, but also by formal arrangements [26]. If part-time working is introduced in a team, a possible team strategy might be to establish formal rules to support individuals in handling daily activities. It is not clear whether part-time work is related to formalisation in medical partnerships. However, some relations with the network structure can be expected. Firstly, the number of individuals in teams with part-timers will increase, because the workload has to be divided between more individuals if some are working part-time. A subsequent formal measure could be to install a formal leader, because of the increasing size of the team.

## Methods

### Selection of teams

Teams of self-employed doctors within three specialties -internal medicine, surgery and radiology- were invited to participate in this study. This invitation was preceded by a questionnaire on the topic of part-time working. We asked for voluntary participation in this team-oriented follow-up study.

The national associations of surgeons (NVH), internists (NIV) and anesthesiologists (NVA) have given permission for the conduct of the study, there are no ethical objections to the study suggested. The national associations supported the study with a written appeal to their members.

Specialties were chosen on the basis of differences in procedures and organisation of work, how they related to the characteristics of their tasks, patient contacts and co-operation with other specialties. Hospitals throughout the Netherlands were represented. As we mentioned the proportion of part-time doctors is still generally low: almost 30% among internists, 28% among radiologists and 18% among surgeons [2].

**Figure 1:**
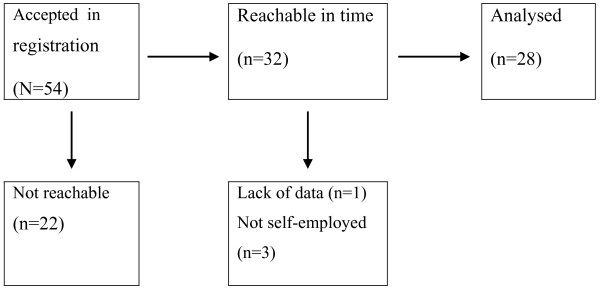
Selection of teams.

Initially 54 teams registered for participation, but only 32 teams could be reached within the research period (Figure 1). The time for observations in hospitals was limited to five months and the summer holiday break interfered with the schedule for visiting complete teams. For analyses, 28 teams were left, after the removal of four teams because data was lacking or because they did not meet the criterion of self-employed partnerships. Integration of part-time work is more difficult for self-employed teams. Therefore we did not include specialists employed by the hospital, because their working arrangements differ markedly.

The number of teams is limited, seven teams of radiologists, ten teams of surgeons and 11 teams of internists. The selection was taken from 85 general hospitals in the Netherlands, where the selected specialties are represented. So our selection was between 8–13% of all specialist teams. Data were collected in 2005.

### Interview and questionnaire

We used data from written individual questionnaires and a semi-structured interview with a representative of each team. The team representatives were visited at the hospital and after the interview the written questionnaires were posted in the hospital for all team members. Within a few days the questionnaires were send back, collected by the team representatives. The response rates were high. 28 interviews and 226 individual questionnaires could be used for analyses as reported in Table 1.

**Table 1:** Response individual questionnaire: team and individual response

	Team response^1^	Individual response		
	Number of teams analysed	Number of questionnaires send	Number of questionnaires returned	Number of questionnaires analysed

Internal medicine	11	142	127	115

Surgery	10	83	77	69

Radiology	7	45	42	42

Total	28	270	246	226

^1 ^Team selection is explained in Figure 1

### Measures

The inventory of network relationships was limited to members of the partnership, collected by the written questionnaire on three different domains: consulting, communication or (intended) relationships of trust [8,9,18]. For each type of relationship the intensity of contacts was also measured. We also asked individually about perceived social-emotional relationships [27].

Furthermore, individual and team characteristics were collected in the interview with a team representative. We asked about individual characteristics such as gender, age, part-time or full-time working, and tenure. The characteristics of the team include its size and formal agreements such as having a formal leader or a number of formal activities, (see team characteristics below) [28].

#### Dependent variables

##### Network characteristics (collected with individual questionnaires)

We asked each individual doctor to answer questions separately about three different types of network. All names of colleagues were mentioned and each individual was asked to mark the colleagues he or she contacted, or would contact, on consulting, communication or (intended) relationships of trust [8,9,18]. Questions for these variables were:

###### 1. For the communication network

Whom among your colleagues do you contact about your work, or things happening at work, and how often?

Scale for the frequency of contacts:

1. A few times a day

2. Every day

3. Almost every day

4. One or two times a week

5. One or two times a month

6. Less often or never

For each relationship in their team individuals scored for the frequency of communication. We aggregated these dyadic scores to the level of individuals by adding the number of contacts on score one – six.

The individual scores on each scale item are expressed in percentages of all contacts in the network. For analyses we divided frequencies of contacts into daily contacts (frequencies of one, two and three as mentioned above) and fewer contacts (frequencies four and five). Score six is seen as not participating in the network. The measure of frequencies of contacts is expressed in percentages of all contacts, divided into a proportion of daily contacts and a proportion of less than daily contacts.

###### 2. For the consulting network

Whom among your colleagues asks your advice and how often? The scale for frequency of contacts was identical to that for communication networks.

###### 3. For the trust network we asked

With whom among your colleagues would you share confidential matters related to work and how intensely would you share such matters?

Scale for intensity:

1. I probably would share confidential matters related to work with...

2. I possibly would share confidential matters related to work with...

3. I certainly would not share confidential matters related to work with...

For each relationship in their team individuals scored for the intensity of trust. We aggregated these dyadic scores to the level of individuals by adding the number of contacts on scores one, two or three. The individual scores on each scale item are expressed in percentages of all contacts in the network. For analyses we divided the strength of the relationship into, 'probably sharing confidential matters' (score 1) and 'possibly sharing confidential matters' (score 2). Score three is seen as not participating in the network. The answers are indicators for actual trust relationships, because they express the intention to share confidential matters. In table 2 score one was used as an indicator for the strength of the relationship of trust.

**Table 2:** Team and individual characteristics

	Teams with part-time workers (n = 20)	Teams without part-time workers (n = 8)	All teams (n = 28)
***Team characteristics***			

	***n***	***n***	***n***

Surgeons	6	4	10

Internists	11	0	11

Radiologists	3	4	7

Teams with formal leader	15	6	21

	***Means***	***Means***	***Means***

Team size^1^	10.3	5.9	9.0

Part-time workers^2^	4.1	-	-

Full-time workers^2^	5.6	5.9	5.6

Men^2^	7.7	5.6	7.1^3^

Women^2^	2.0	0.1	1.4^3^

Nr. of formal activities	5.5 (1.3)	5.0 (0.9)	5.4 (1.2)

Average fte	0.90^a^	1.0	0.97


***Individual characteristics***			

	***N***	***N***	***N***

Women	38	1	39^3^

Men	143	42	185^3^

Part-time	77	-	77

Full-time	105	44	149

	***Means***	***Means***	

Age	48.1 (7.9)	49.1 (7.5)	48.3 (7.8)

Tenure	9.4 yrs (8.4)	9.3 yrs (7.0)	9.4 yrs (8.2)

^1 ^actual team size including non-responding individuals

^2 ^based on responding individuals

^3 ^gender is unknown for 2 individuals

^a ^average of part-time working doctors separately = 0.76

### Aggregated network measures

Gathering data within teams gave information at the level of networks of individual doctors, but also at the level of the entire team. Relationships within the entire team are called the full network. Relationships within the network of an individual doctor are called personal or ego networks. In this study we use data from both levels, but the central questions focus on aspects of personal networks. We want to trace differences between fulltime and part-time doctors which can be found at the level of ego networks only.

Software for network analyses of the UCInet package [29] was used. From the SPSS-database the network data on consultation, trust and communication networks were imported in UCInet. We used two variables from secondary measures based on UCInet calculations, and their definitions are [30]:

- *Size *of the ego-networks is the total number of all direct (one-step) relationships of ego, plus ego itself. ('Ego' is used for the individual or the actor of the personal network.)

- *Reach efficiency *gives the percentage of all secondary contacts divided by size. It expresses how many (non-redundant) secondary contacts ego gets for each unit of primary contact. Reach efficiency is high if direct relations have a lot of unique contacts, which cannot be reached directly by ego.

#### Independent variables

##### 1. Individual characteristics

In this study we used characteristics of ***individual ***doctors: age, gender, part-time working (< 1.0 fte) or full-time (1.0 fte), years in partnership (tenure). The division between part-time and full-time working was based on self-reported formal participation expressed in full-time equivalents (fte). Working less than 100% (1.0 fte) is defined as working part-time.

##### 2. Team characteristics

Formal structural aspects were measured by characteristics of the ***team ***such as team size and the degree of formalisation calculated by adding up the number of formal activities to a maximum of eight. These include the following activities: using guidelines, having joint medical policy, using electronic communication, using electronic patient records, using specialised support (physician assistants), using week schedules and year planning, having internal rules and finally, having a formal leader. In the analyses having a formal leader was included separately.

Furthermore, perceived social and/or emotional team relationships were measured. The individual scores were aggregated to the team level as a measure of team climate. A scale developed by Stogdill & Bass [27] was used. An example item is: "The climate in our team is relaxed" (5-point scale; Crohnbach's Alpha .86).

### Testing the hypotheses with multilevel analyses

Doctors working in a team are similar with regard to team characteristics. Multilevel analysis is used to analyse hierarchically structured data: individual medical specialists are nested in teams [31,32]. With multilevel analysis total variation in dependent variables of personal network structure, such as size and reach efficiency, is divided into one part due to differences between doctors, and one part due to differences between teams. In multilevel modelling the dependency structure in data is taken into account. Networks of individual team members are not independent and therefore no simple OLS regression analyses can be estimated. For the analyses the MLwiN software package was used [33].

### Modelling strategy

Three models were analysed. Firstly, a reference model, including only team size, because individual networks cannot be compared without taking team size into account. Team size is basically the opportunity structure for contacts. In the second model the individual characteristics are added: being a part-time worker, gender and age. Correlations between variables in our model were inspected and 'tenure', being highly correlated to age, was not used in analyses. In the third model team characteristics are added: having a formal leader, the degree of formalisation, positive team relations, a dummy variable for specialty and finally teams with part-timers, as opposed to full-timer teams. To prevent over-identification of team characteristics, we added and removed each team variable separately in analyses, because the number of cases is small at the second level (n = 28). Obviously, the effects of a few characteristics were related, like having a formal leader, the degree of formalisation and positive team relations. These effects are not due to over-identification, but are related to content. Adding separate team variables to the analyses did not bring up unexpected and different effects compared to adding all team variables in one step. Therefore, we decided to present this full model in our tables.

As a last step, single level and cross-level interactions were analysed. We included these effects in the presentation of the full model.

## Results

### Team and individual characteristics

Our study focuses on teams *with and without part-time working medical specialists*. The first type of team is mostly mixed, containing both part-time and full-time doctors. Two small teams with part-timers only were also included in this first type of team.

Table 2 gives characteristics of both types of teams. Most teams with part-time workers consisted of internal medicine teams and in this specialty no teams without part-time workers are included. In surgery and radiology both types of teams are included.

The majority of both types of teams has a formal leader and the number of formal activities is on average five to six activities out of eight. Teams with part-time workers are larger. In teams with part-timers both more men and women are found. Age (48–49) and tenure (over 9 years) is in both types of teams almost equal. With one exception all female specialists work in teams with part-timers and actually only one female doctor worked full-time.

### Differences in network characteristics of part-time and full-time workers

Aspects of the personal network structures were compared in all three types of networks: consulting, communication and trust. Multilevel analyses were used to compare means and we corrected for team size. We divided the population of full-time workers into two groups: full-timers in a team with part-time colleagues and full-timers in a team with full-time workers only. Table 3 shows the differences in informal work-related network characteristics of these two groups – full-timers compared to part-timers.

**Table 3:** Differences between part-time workers (PT) and full-time workers (FT) in characteristics of ego networks, controlling for team size (two groups of full-time workers :*in teams with part-time workers and in teams with full-timers only*)

	Part-time workers *(N = 77)*	Full-time workers in team with PT workers *(N = 105)*	Full-time workers in team with FT workers only *(N = 44)*	***Difference ***Pt -Ft in Pt-team	***Difference ***Pt -Ft only	***Difference ***Ft in Pt-team – Ft only
***Ego network characteristics***	***Means (SD)***	***Means (SD)***	***Means (SD)***	***p-value***	***p-value***	**p-value**

**Consulting networks**						

Network size	6.51 (0.4)	6.35 (0.4)	5.24 (0.6)	.60	.09	.13

Reach efficiency	21.10 (2.3)	23.20 (2.3)	30.10 (3.7)	**.02**	**.05**	.14^2^

< Daily contacts^a^	60.61 (7.1)	64.10 (7.0)	51.68 (7.2)	.48	.21	.09


**Communication networks**						

Network size	7.68 (0.3)	7.36 (0.3)	7.30^1 ^(0.4)	.16	.44	.91

Reach efficiency	17.37 (1.7)	17.81 (1.7)	22.68 (2.8)	**.04**	.12^2^	.16^2^

< Daily contacts^a^	40.49 (5.4)	40.63 (5.4)	22.12 (9.1)	.97	**.04**	**.04**


**Intended trust networks**						

Network size	7.75 (0.2)	7.53 (0.2)	7.47^1 ^(0.3)	.16	.51	.88

Reach efficiency	17.18 (1.7)	17.56 (1.7)	22.29 (2.7)	**.02**	.12^2^	.15^2^

Probably sharing confidential matters^a^	68.45 (9.0)	68.88 (8.9)	69.02 (7.9)	.98	.94	1.00

Compared in multilevel analyses, with explicit pair wise tests

^a ^expressed in percentages of all contacts

^1 ^ego-network size is high in comparison with team size as a consequence of relatively more respondents in large teams, which increases the average here (and the correction for team size has some impact)

^2^significant differences were expected, but not found in pair wise explicit testing, which might by caused by the low N of full-timers in teams with full-timers only.

#### Size of the personal network

Firstly, the size of all three types of personal networks did not differ between part-time and full-time doctors, whereas we expected part-timers to have smaller networks.

#### Reach efficiency

In all three network types reach efficiency differs between part-timers and full-timers in mixed teams. In consulting networks, this difference is also seen between part-timers and full-timers in teams with full-timers only. Contrary to what was expected reach efficiency of part-timers is lower in comparison to full-timers (Table 3).

#### Frequency

In *communication *networks a low frequency in contacts, amounting to less than once a day, is found for part-timers and full-timers in mixed teams. They differ, however, on this point with full-timers in full-time teams. In *consulting *networks, no differences in frequency of contacts were found. In general frequency of contacts is higher in communication compared to consulting relationships (in Table 3).

#### Strength

As expected the indication for *trust *contacts expressed as an intention to share confidential matters is not different for part-timers and full-timers. Intended trust relations are found for almost 70% of all doctors (Table 3).

### Testing the hypotheses: the relationship between part-time working and social networks

To answer our central question, we examined the impact of part-time working and other individual and team characteristics on characteristics of networks: size, reach efficiency and frequency or strength. Firstly, inspecting our reference model, we found significant variation between teams in personal network size and reach efficiency, for all three types of networks. This means that size (Table 4) and reach efficiency (Table 5) differ between teams, whether it concerns communication, consultation or trust networks. In frequency or strength of relations (Table 6) significant variation between teams is only found in communication networks. Secondly, looking at the intra-class correlation of the reference models, it turns out that variation in reach efficiency is mainly at the team level. For the size of the intended trust and consulting networks variation is more or less equally divided between the individual and the team level. For the size of the communication networks and frequency, or strength, of all three types of networks, variation is mainly at the individual level. To find out what specific characteristics in our model influence the network aspects we will inspect the data more closely.

**Table 4:** Relationship of individual and team characteristics with the size of ego networks in consulting, communication and intended trust relations

	**SIZE consulting**			**SIZE communication**			**SIZE intended trust**		
	Model 0 estimate		Full model estimate	Model 0 estimate		Full model estimate	Model 0 estimate		Full model estimate

Constant	6.09 (0.3)*	6.11 (0.3)*	6.15 (0.3)*	7.45 (0.2)*	7.46 (0.2)*	7.41 (0.1)*	7.58 (0.2)*	7.59 (0.2)*	7.55 (0.2)*

***Corrected*** ***for:***									

Team size	0.38 (0.07)*	0.38 (0.06)*	0.29 (0.08)*	0.76 (0.04)*	0.75 (0.04)*	0.78 (0.04)*	0.82 (0.04)*	0.82 (0.04)*	0.82 (0.04)*

***Individual*** ***characteristics:***									

Part-time worker		0.06 (0.3)	-0.01 (0.3)		0.24 (0.2)	0.19 (0.3)		0.23 (0.2)	0.20 (0.2)

Female		0.19 (0.4)	0.19 (0.4)		0.27 (0.3)	0.31 (0.3)		-0.09 (0.2)	-0.07 (0.2)

Age		-0.04 (0.02)*	-0.04 (0.02)*		0.002 (0.01)	0.004 (0.01)		-0.02 (0.009)*	-0.02 (0.009)*

***Team*** ***characteristics:***									

Formal leader (yes = 1)			0.58 (0.7)			0.91 (0.3)*			0.98 (0.4)*

Number of formal arrangements			0.28 (0.3)			-0.06 (0.1)			-0.09 (0.1)

Team with PT-workers			0.96 (0.8)			0.40 (0.4)			0.35 (0.4)

Surgeon^1^			-0.63 (0.9)			-0.57 (0.4)			0.35 (0.4)

Radiologist^1^			-0.51 (0.9)			-0.41 (0.4)			-0.37 (0.4)

Positive team relations			0.07 (0.5)			0.04 (0.2)			0.19 (0.2)

Team size*formal leader						0.28 (0.06)*			


Team variance	2.17 (0.7)*	2.11 (0.7)*	1.79 (0.6)*	0.76 (0.3)*	0.73 (0.3)*	0.17 (0.1)	0.69 (0.2)*	0.65 (0.2)*	0.44 (0.2)*

Individual level variance	2.87 (0.3)*	2.77 (0.3)*	2.84 (0.3)*	1.87 (0.2)*	1.87 (0.2)*	1.90 (0.2)*	0.83 (0.08)*	0.82 (0.08)*	0.84 (0.09)*

Intra-class correlation	43,1%	43,2%	38,7%	28,9%	28,1%	8,2%	45,4%	44,2%	34,4%

^1 ^internist as the reference category

**Table 5:** Influence of individual and team characteristics on reach efficiency of ego networks in consulting, communication and trust intended relations

	**REACH EFFICIENCY in consulting**			**REACH EFFICIENCY in communication**			**REACH EFFICIENCY in intended trust**		
	Model 0 estimate		Full model estimate	Model 0 estimate		Full model estimate	Model 0 estimate		Full model estimate

Constant	24.49 (1.9)*	24.41 (1.9)*	24.51 (1.9)*	19.06 (1.5)*	19.05 (1.5)*	19.39 (1.3)*	18.79 (0.4)*	18.78 (1.0)*	19.12 (1.3)*

***Corrected*** ***for:***									

Team size	-0.60 (0.4)	-0.59 (0.4)	-0.15 (0.5)	-1.67 (0.3)*	-1.66 (0.3)*	-1.35 (0.4)*	-1.79 (0.3)*	-1.78 (0.3)*	-1.51 (0.3)*

***Individual*** ***characteristics:***									

Part-time worker		-1.81 (1.0)	-1.86 (1.0)		-0.39 (0.2)	-0.41 (0.25)		-0.37 (0.177)*	-0.68 (0.2)*

Female		-0.28 (1.1)	-0.19 (1.2)		-0.19 (0.3)	-0.12 (0.3)		0.06 (0.2)	0.10 (0.2)

Age		0.16 (0.05)*	0.16 (0.05)*		0.008 (0.01)	0.009 (0.01)		0.02 (0.009)*	0.02 (0.009)*

Team size*part-time worker									0.08 (0.04)*

***Team*** ***characteristics:***									

Formal leader (yes = 1)			-3.31 (4.6)			-3.03 (3.3)			-2.75 (3.5)

Number of formal arrangements			-0.97 (1.9)			0.48 (1.4)			0.58 (1.3)

Team with PTworkers			-7.53 (5.3)			-6.48 (3.7)			-6.56 (3.5)

Surgeon^1^			-0.67 (5.5)			-2.45 (4.0)			-3.43 (3.8)

Radiologist^1^			3.53 (5.7)			6.00 (4.0)			5.40 (3.8)

Positive team relations			-0.11 (3.1)			-0.49 (2.2)			-0.61 (2.1)

									

Team variance	101.69 (28.1)*	96.77 (26.8)*	86.95 (24.8)*	59.46 (15.9)*	56.61 (15.7)*	46.37 (12.7)*	55.67 (14.9)*	54.83 (14.7)*	41.84 (11.4)*

Individual level variance	28.67 (2.9)*	26.85 (2.7)*	27.42 (2.8)*	1.54 (0.2)*	1.52 (0.2)*	1.56 (0.2)*	0.87 (0.09)*	0.84 (0.09)*	0.83 (0.09)*

Intra-class correlation	78,0%	78,3%	76,0%	97,5%	97,4%	96,7%	97,6%	98,5%	98,1%

^1 ^internist as the reference category

**Table 6:** Influence of individual and team characteristics on the frequencies in contacts in consulting and communication relations and strength of ego network in intended trust relations

	**< DAILY CONTACTS in consulting**			**< DAILY CONTACTS in communication**			**STRONGEST TRUST indication**		
	Model 0 estimate		Full model estimate	Model 0 estimate		Full model estimate	Model 0 estimate		Full model estimate

Constant	59.55 (2.8)*	58,82 (2.9)*	58.20 (2.9)*	35.33 (3.8)*	35.47 (3.8)*	35.38 (2.3)*	68.77 (3.8)*	69.55 (3.0)*	68.27 (2.2)*

***Corrected*** ***for:***									

Team size	-1.67 (0.6)*	-1.49 (0.6)*	-2.53 (0.7)*	2.70 (0.8)*	2.69 (0.8)*	1.04 (0.6)	-2.68 (0.6)*	-2.82 (0.6)*	-2.34 (0.5)*

***Individual*** ***characteristics:***									

Part-time worker		-3.16 (5.0)	-5.93 (5.3)		-2.14 (4.5)	-1.74 (4.5)		-1.75 (4.8)	-0.55 (4.9)

Female		3.63 (4.4)	3.91 (6.3)		-3.21 (5.4)	-2.93 (4.5)		4.12 (6.5)	5.86 (6.0)

Age		-0.65 (0.3)*	-0.66 (0.3)*		-0.33 (0.2)	-0.26 (0.2)		0.46 (0.3)	-0.50 (0.3)

***Team*** ***characteristics:***									

Formal leader (yes = 1)			1.42 (7.2)			22.85 (5.9)*			-11.24 (5.4)*

Number of formal arrangements			0.85 (2.7)			-4.89 (2.2)*			1.74 (1.9)

Team with PTworkers			15.44 (8.5)			14.98 (7.0)*			11.55 (6.5)

Surgeon^1^			-1.94 (8.0)			-7.44 (6.5)			14.96 (5.8)*

Radiologist^1^			-16.25 (8.5)			-25.47 (6.9)*			0.54 (6.2)

Positive team relations			0.24 (4.4)			-7.15 (3.5)*			12.74 (3.1)*

									

Team variance	73.82 (52.0)	102.98 (59.2)	77.32 (50.7)	310.08 (107.5)*	297.52 (104.2)*	43.34 (33.3)	16.12 (11.5)	122.66 (62.1)	0.0 (0.0)

Individual level variance	898.19 (91.1)*	850.26 (87.0)*	799.68 (82.8)*	585.00 (59.4)*	589.56 (60.4)*	596.24 (61.6)*	202.58 (20.5)*	775.95 (79.3)*	760.21 (73.8)*

Intra-class correlation	7,6%	10,8%	8,8%	34,6%	33.5%	6,8%	13,7%	13,6%	0%

^1 ^internist as the reference category

#### The relationship with size

In all three types of networks, neither working as a part-time doctor, nor working in a team with part-timers has an influence on the **size **of informal work-related ego networks (Table 4). This means that our first hypothesis is not confirmed. Part-timers' networks are not smaller, compared to full-timers' networks.

Not surprisingly, in all three types of networks, a larger team size was related to larger personal work- related networks. Furthermore, the younger the doctors are, the larger their personal network is in consulting and intended trust relations. If a team had a formal leader, personal communication and intended trust networks were larger. But apart from team size and a formal leader, no influence of further team characteristics was found. Finally, in communication networks an interaction effect was found between team size and a formal leader, meaning that communication networks of doctors are larger if teams are bigger and have a formal leader (Table 4).

#### The relationship with reach efficiency

Contrary to expectations (hypothesis 2), neither part-time doctors individually, nor teams with part-timers have a higher **reach efficiency **in all three types of networks (Table 5). Unexpected reach efficiency was higher for full-time and older doctors in small-sized networks of intended trust relationships. Interesting is that the interaction effect here points out that in trust relationships, if teams are also larger for part-time doctors, reach efficiency is high. However this interaction effect is rather small. In consulting relations older doctors have higher reach efficiency and in communication networks a small team size was related to reach efficiency.

Although the ability to reach efficiently, what we call efficient "reachability", differs between teams those differences could not be explained by team variables in our model, with the exception of the team size. So, team characteristics which are not included in this study are important for explaining efficient reachability (Table 5).

#### The relationship with intensity

Several team characteristics influenced intensity in all three types of networks. (Table 6).

In communication networks we found as expected that in teams with part-timers contact frequencies are lower, compared to full-time teams. So, hypothesis one was confirmed for communication networks: in teams with part-timers doctors see each other less often.

Furthermore, if teams have a formal leader and if the degree of formalisation is low, they contact each other less for communication. Positive team relations are related to seeing each other more often to communicate. And finally, the specialty of doctors is important for contact frequency in communication as well as in intended trust relations.

In consulting networks contact frequency is higher if teams are smaller. Especially younger doctors have a higher frequency in consulting contacts.

In hypothesis three, the strength of intended trust relations was, as expected, not related to part-time working, neither individually, nor for teams. The strongest intention for trust was mainly found first in small teams, then additionally in teams without a formal leader and finally in teams with positive team relations. Especially in teams with surgeons the intentions for trust relations were strong (Table 6).

## Discussion and conclusion

In answer to the main question, we conclude that part-time working does not have a great effect on informal work-related networks of doctors. In two points our hypotheses are confirmed: the frequency of communication contacts is lower in mixed teams, compared to full-time teams and the strength of intended trust relationships is equally high for part-timers and full-timers. Below we will discuss our findings in more detail and give our conclusions.

### The meeting argument and time restrictions

In this study part-timers' time restrictions only influence how often doctors in teams with part-timers contact each other. The size of their networks is as high as full-timers' networks (hypothesis 1). This finding is in contrast with earlier research [34] stating that part-time workers are less focussed on investments in work relations. A plausible explanation could be that teams of doctors are not that large (5–20 individuals) and that part-time working doctors are still working a large part of the week (0.76 fte), so they have sufficient opportunities to meet their colleagues.

As expected a lower frequency in contacts is found for part-time working doctors. In earlier research [17] was found that working time was related to the strength of the relationships. In this line of reasoning it would be interesting to find out whether a lower frequency in contacts would affect the quality of relationships in terms of professional support or the transfer of expertise.

### Reach efficiency and social capital

Reach efficiency was not high for part-time doctors. We expected that part-timers would seek colleagues who have many contacts in order to ensure the best possible chance of receiving information (hypothesis 2). But our finding is that efficiency in managing relations seems not to work for part-time doctors. This finding is in line with a relatively large network size for part-time doctors. Obviously, part-timers are not aiming at efficiency by limiting the size of networks or by choice of specific relations, e.g. non redundant reachability. In terms of social capital we can value the investments of part-time doctors positively, because the basis of their resources -the size of networks- is comparable to full-time doctors' resources. The question is whether part-time doctors build relations with the most resourceful individuals for their goals at work. Burt [20] refers to those resourceful individuals as bridging colleagues. Those bridging network members link subgroups in networks and have the advantage of control over information. So, in terms of efficiency, part-time doctors should contact bridging colleagues. But our findings are different. Part-time doctors might want to contact their colleagues themselves, because any information given indirectly can be changed, influenced or incomplete. In terms of quality demands and prevention of communication errors, part-time doctors might by carefully trying to get their information as directly as possible. In this line of reasoning the interaction effect we found for intended trust relationships can be understood, implying that with the growth of teams reach efficiency of part-time doctors is higher. Hence, if a team becomes larger part-time doctors will not be able to maintain all their intended trust contacts directly.

It is intriguing that differences in reach efficiency between teams were only influenced by team size and no further team characteristics explained those differences. Maybe bridging individuals who have control over information play an important role. Burt [20] found that these colleagues were the bridges over so-called structural holes in their networks, which gave them disproportionate say in whose interests are served when contacts meet. This brings up the subject of structural holes which might be interesting to investigate in medical teams.

### Balancing between low frequencies in contacts and growing formalisation

We found that lower contact frequencies in communication networks are found in teams with a formal leader. Furthermore an interaction effect was found, meaning that communication networks are larger if teams grow and have a formal leader. These findings are in line with the plausible idea that formal leadership will be needed together with the growth of part-time members and consequently larger teams [28]. However, other formalisation measures decreased in larger teams. It can be argued that having a formal leader might replace other formal measures like formal team meetings. Another realistic argument is that recent hospital mergers caused teams to grow larger. In those situations new larger teams have not yet developed joint formal rules. So generally, formalisation can be an efficiency strategy, beneficial for larger teams. In our study this relationship was limited to a formal leader in larger teams. This can also explain to some extent why part-time doctors are not individually aiming at efficiency, because a formal team leader can be an adequate alternative for information needs.

Furthermore, it was not surprising to find that frequent, perhaps daily, communication contacts go together with positive team relations. Positive team relationships were also found if the intended confidential relationships were strong. But contrary to the impact of formal leadership in communication networks the intended confidential relationships were not related to having a formal leader. These findings indicate that a formal leader can compensate for limited opportunities for doctors to talk about their work, but not for sharing confidential matters.

### Limitations of this study

A basic limitation of this study is the number of teams selected. Data were gathered from 28 teams of physicians, which is a small part of all the medical specialist teams in the Netherlands. But as we argued in the method section, the selected teams were differentiated with regard to the dominant characteristics of specialties and their location in the Netherlands. Some tendencies, as we found in interaction effects, might have been more explicit if we had a larger sample with more variation on some crucial factors, such as team size. Furthermore, it would be interesting if we could have made more subgroups to compare, e.g. the comparison of teams with just a few part-time workers against teams with a large number of part-timers. However, the number of teams with those characteristics was too small. The same argument holds for the gender comparison, since female doctors are actually almost all part-timers.

Questions on the quality of relationships in networks came up in our discussion. These questions point to further exploration at the level of dyadic relations. This study was limited to the individual and team level. Analyses at the level of dyads would give more insight into the preferences of medical specialists in their relations. Are part-time doctors more related to other part-time workers in their team, compared to relations with full-time colleagues? Are the most intense trust relationships found more among part-time and full-time colleagues separately in a team? Are communication, consultation and trust relationships built with the same colleagues, or not? Are some colleagues bridging individuals between subgroups? It would be interesting to explore these issues at the level of dyads.

Another point is that our study was limited to work-related networks of doctors, which might to some extent explain why our findings are different from earlier research at some points. Many empirical studies on networks are focussed on families and neighbourhoods, which differ from the work context. And even if studies focussed on networks in work situations they differed markedly from the networks of doctors (e.g. networks of managerial boards). So, our references to other studies are only partly comparable.

### Implications for part-time working in medicine

Part-time working seems not to affect highly informal networks in medical teams. From this basic conclusion we can derive two different lines of reasoning: Firstly, on the role networks play in the lives of part-time doctors, and secondly, on the importance of informal networks as a strategy in structuring work situations in teams with part-timers.

For professionals, such as doctors, work seems to be a very important part of their lives, even if they work part-time [35]. We might conclude that part-time working doctors want to avoid an exceptional position in their team. Part-time physicians are still working 40 hours a week [36], which is a regular full-time week in other professions. In this study we found that part-time doctors have comparable network sizes as full-timers, although they spend fewer hours at the hospital. The same line of reasoning can be followed on the subject of efficient reachability. An efficient involvement in network relationships could be risky for part-timers, because they might find themselves in an isolated position. The risk of isolation would be even higher if part-timers would invest less in trust relationships, therefore missing important partnerships' decisions. So, informal work-related networks are very important for part-time doctors in order to maintain their position in the team.

Our second point here is focussed on the way teams with part-time doctors structure their co-operation. Part-time and full-time doctors invest equally in networks. However, it seems reasonable that this strategy can only be maintained so long as teams are relatively small. The tendency that with the growth of the size of teams, part-timers can not maintain their basic strategy was found in this study. The influence of formal leadership was related to a larger team size.

From these findings we can conclude that solutions for organisational difficulties in co-operation between full-time and part-time doctors are found in some aspects of formalisation, especially through a formal leadership. A formal leader might compensate for the traditional ways of communication within informal networks, which are less adequate if part-time working increases.

## Competing interests

The authors declare that they have no competing interests.

## Authors' contributions

PH performed the statistical analyses, drafted the manuscript and contributed to all other aspects of the study, JdJ participated in the critical revision of the manuscript, PG participated in the design of the study and the critical revision of the manuscript, LH contributed to the acquisition of data and was involved in drafting the manuscript, BV participated in the critical revision of the manuscript and PS contributed as statistical consultant. All authors have given final approval of the submitted manuscript.

## Pre-publication history

The pre-publication history for this paper can be accessed here:



## References

[B1] De Jong JD, Heiligers Ph, Groenewegen PP, Hingstman L (2006). Why are some medical specialists working part-time, while others work full-time?. Health Policy.

[B2] Heiligers PJM, De Jong JD, Hingstman L, Lugtenberg M, Groenewegen PP (2006). Integratie deeltijdwerken chirurgen: Meningen over de organisatie van deeltijdwerken en arbeidsproduktiviteit van maatschappen met en zonder deeltijders. [Integration of part-time working surgeons: opinions about the organization of part-time work and production of partnerships with and without part-timers).

[B3] Heiligers Ph, Hingstman L (2000). Career preferences and the work-family balance in medicine: gender differences among medical specialists. Social Science & Medicine.

[B4] McMurray JE, Heiligers PJM, Shugerman RP, Douglas JA, Gangnon RE, Voss C, Costa ST, Linzer M (2005). Part-time medical practice: Where is it headed?. The American Journal of medicine.

[B5] Velden LJF Van der, Bennema-Broos M, Hingstman L (2001). Monitoring arbeidsmarkt chirurgen. [Monitoring workforce of surgeons].

[B6] Van Lier M, Ten Hoopen L, Verheij F (2004). Van nood tot deugd. Anticiperen op deeltijdwerk medisch specialisten. [From emergency to virtue Anticipating on part-time working medical specialists] Medisch contact.

[B7] Lens P, Wal G Van der, eds (1997). Problem doctors: a conspiracy of silence.

[B8] Flap HD, Völker BGM, eds (2004). Creation and returns of social capital: a new research program.

[B9] Lazega E (2001). The collegial phenomenon.

[B10] Van Emmerik IJH (2006). Gender differences in the creation of different types of social capital: A multilevel study. Social Networks.

[B11] Blau PM (1977). Inequality and heterogeneity. A primitive theory of social structure.

[B12] Flap H, Völker B (2001). Goal specific social capital and job satisfaction. Effects of different types of networks on instrumental and social aspects of work. Social Networks.

[B13] Granovetter MS (1973). The strength of weak ties. American Journal of sociology.

[B14] Flap H, Kalmijn M (2001). Assortative Meeting and Mating: Unintended Consequences of Organized Settings for Partner Choices. Social forces.

[B15] Feld S (1981). The focused organization of social ties. American Journal of Sociology.

[B16] Poel MGM Van der (1993). Personal networks: a rational choice explanation of their size and composition.

[B17] Sanders K, Van Duijn M (2001). Sociale cohesie binnen organisaties: frequentie van informele contacten en sterkte van bindingen. [Social cohesion within organizations: frequencies of informal contacts and strength of ties] Sociale Wetenschappen.

[B18] Krackhardt D, Hanson JR (1993). Informal Networks: The Company behind the Chart. Harvard Business Review.

[B19] Groenewegen PP, Hansen JJHH, ter Bekke S (2007). Professies en de toekomst: veranderende verhoudingen in de gezondheidszorg. [Professions and the future: changing relations in health care].

[B20] Burt RS (2002). Bridge decay. Social networks.

[B21] Dekker RJ (2000). *De wetenschappelijke match: persoon-cultuurfit van loopbanen van mannelijke en vrouwelijke wetenschappers.*. [The scientific match: person-culture fit in careers of male and female scientists].

[B22] Moor G (2000). Structural determinants of men's and women's personal networks. American Sociological Review.

[B23] Straits BC (1996). Ego-net diversity: same- and cross-sex coworker ties. Social networks.

[B24] Tijhuis MAR, De Jong-Gierveld J, Feskens EJM, Kromhout D (1999). Changes in and factors related to loneliness in older men. The Zutphen Elderly Study. Age and ageing.

[B25] Zenger TR, Lawrence BS (1989). Organizational demography: the differential effects of gender and tenure distributions on technical communication. Academy of Management Journal.

[B26] Podolny GM, Baron JN (1997). Resources and relationships: social networks and mobility in the workplace. American Sociological Review.

[B27] Stogdill RM, Bass BM (1990). Bass & Stogdill's handbook of leadership: theory, research and managerial applications.

[B28] Kalleberg AL (2000). Nonstandard employment relations: part-time, temporary, and contract work. Annual Review of Sociology.

[B29] Borgatti SP, Everett MG, Freeman LC (2002). Ucinet for Windows: Software for Social Network Analysis.

[B30] Hanneman RA, Riddle M (2005). Introduction to social network methods.

[B31] Snijders TAB, Bosker RJ (1999). Multilevel Analysis. An introduction to basic and advanced multilevel modeling.

[B32] Leyland AH, Groenewegen PP (2003). Multilevel modeling and public health policy. Scand J Public Health.

[B33] Goldstein H (1995). Multilevel statistical models.

[B34] Van Dyne L, Ang S (1998). Organizational citizenship behavior of contingent workers in Singapore. Academy of Management Journal.

[B35] Soethout MBM, Ten Cate TJ, Wal G Van der (2004). Factors associated with the nature, timing and stability of the specialty career choices of recently graduated doctors in European countries. Med Educ Online [serial online].

[B36] De Jong JD, Heiligers Ph, Groenewegen PP, Hingstman L (2006). Part-time and full-time medical specialists, are there differences in allocation of time?. BMC Health Services Research.

